# The Removal of the Upper Maxillary Nerve at the Foramen Rotundum

**Published:** 1892-12

**Authors:** M. Storrs

**Affiliations:** Hartford


### THE REMOVAL OF THE UPPER MAXILLARY NERVE AT THE FORAMEN ROTUNDUM.

By M. STORRS, M. D., of Hartford.

[From Transactions of the Tenth International Medical Congress, Berlin, 1890.]

The operation which I am about to describe is the division of the second branch of the trifacial nerve at the foramen rotundum, and its entire removal. It does not necessarily include the removal of Meckel's ganglion, but the removal of the nerve beyond the ganglion divides the posterior dental nerve and the spheno-palatine branches.

*Operation.*—The nerve is removed in the following manner: A curvilinear incision is made along the lower border of the orbit through the lid. The periosteum is divided and lifted from the floor of the orbit, the eye being held up by a spoon-shaped spatula, the handle bent at an angle of ninety degrees. The nerve is laid bare in the orbital groove; sometimes it is found covered with a thin layer of bone. If there should be any difficulty in finding the nerve, a probe can be passed through the infra-orbital foramen, and be carried into the orbit. The nerve is then lifted from the groove with a small strabismus-like hook, and a ligature is placed around it, about three-quarters of an inch from the edge of the orbit. This ligature gives full control of the nerve. It can be stretched, or changed in its direction, having already been divided externally to the ligature.

Using a small, thin, narrow, curved, dental spatula, the end perhaps serrated, the dissection can be carried backward to the foramen rotundum, separating the nerve from all the other tissues. Should the spheno-maxillary fissure be too narrow for entrance into the fossa, which happens when the antrum is high, it can be depressed by pushing a wedge of steel, something like a screw-driver, under the sphenoid bone, and depressing the floor of the orbit downward and inward. No harm is done in this depression.

The deeper dissection divides or separates the posterior dental and spheno-palatine branches from the maxillary nerve. The instrument feels the resistance of the bone when the foramen is reached, just about two inches from the edge of the orbit. The ligature, holding the nerve, is now drawn through a wire loop or snare. This needs to be made with great care; otherwise the nerve will be drawn down into the canula, and it is not, from its strong and elastic character, divided. The one that I have used for this purpose is a small steel canula, slightly curved, flattened at the end, and sharpened. The loop is pushed backward by the spatula already spoken of, and when it reaches the foramen, it is tightened and the nerve is divided. The portion of the nerve controlled by the ligature is now removed, about one inch. By a slight dissection downwards from our first incision, the nerve in the infraorbital foramen is now drawn out by gathering up with a hook the mesh of branches going to the cheek. The nerve is then put into a loop of a threaded needle, and carried down into the mouth, leaving the end which had been divided in the middle of the orbit suspended between the upper alveolus and the cheek. The divided ends of the nerve are now some three inches apart, and the ends are no longer opposed to each other. This reversing of the distal portion was first suggested by the late Dr. Hodgen, of St. Louis. This bit of surgical strategy makes a reunion impossible. The operation is done antiseptically, and is completed by putting catgut drainage into the groove passing out through the orbital foramen, and from there carried either directly through the cheek or made to follow the nerve down into the mouth. The wound is then closed.

*Remarks.*—This operation is made very simple and easy (1) by the depressing of the floor of the orbit, thus affording ample room; (2) by the ligature controlling the nerve, which, when stretched, becomes a guide to the foramen rotundum; and (3) by the wire loop, which cannot injure other structures. Again, there is no disfiguring of the face in this operation, and neither can any of the deep-seated parts receive any injury from the loop. It cannot wound the terminal branches of the internal maxillary in the fossa, and in the orbit all the parts are above the place of our operation; as the optic nerve passes through the optic foramen, the second, third, and the ophthalmic branch of the fifth nerve pass through the sphenoidal fissure. The muscular attachments are also all above. All the inconvenience arising from the loss of this nerve is the impairment of sensation.

*Results.*—My first operation was done four years ago, and there has been no return of the neuralgia. The relief was so perfect, and the operation so slight, that the patient, a man aged seventyfive, declared that, if it were necessary, he would have the operation done every week. Two subsequent cases have been alike successful. When failure has resulted after the various attempts to remove this nerve, it may be due (1) to the division of the nerve on the distal side of the posterior dental nerve, which, we believe, from its distribution is more frequently the cause of trouble than the sphenopalatine branches going to Meckel's ganglion, or (2) to intercranial disease which might have been more accurately diagnosticated, or (3) to a restoration of the exsected nerve. We cannot absolutely determine how far the nerve, whose ends are left in a long channel, might reach to unite, but the great displacement of the ends of the nerve, when reversed, makes a reconstruction impossible.

